# Annual and Analytical Cyclopaedia of Practical Medicine

**Published:** 1900-03

**Authors:** 


### Books and Magazines.

---

Annual and Analytical Cyclopaedia of Practical Medicine. By Charles de M. Sajous, M. D., and one hundred associate editors, assisted by corresponding editors, collaborators and correspondents. Illustrated with chromo-lithograph engravings and maps. Volume IV. Philadelphia, New York and Chicago: The F. A. Davis Co., publishers. 1899.

This book takes up each disease in a general article, and sustains it by the solvent points of the literature of the last ten years. The plan is an excellent one, as it places before the reader in convenient arrangement the whole subject. The articles are written by experts, the type is large and clear, and the columns doubled, which make the reading easy and convenient.

The late Dr. George H. Rohe wrote the article on "Insanity," which clearly elucidates the subject in all of its modern bearings. Dr. Blockader, of Montreal, contributed the paper on "Diarrheal Diseases of Infants," which will be read with much profit during the coming summer. There is a most excellent article on "Malarial Fevers," by James C. Wilson and Thos. G. Ashton. The other papers are by the best men, and are on subjects equally practical. This work is meeting with great favor, which is encouraging to the editors and publishers. The price is not mentioned in the volume at hand. T. J. B.

